# Sleep Disruption and Proprioceptive Delirium due to Acetaminophen in a Pediatric Patient

**DOI:** 10.1155/2013/471294

**Published:** 2013-03-18

**Authors:** Carla Carnovale, Marco Pozzi, Andrea Angelo Nisic, Elisa Scrofani, Valentina Perrone, Stefania Antoniazzi, Emilio Clementi, Sonia Radice

**Affiliations:** ^1^Unit of Clinical Pharmacology, Department of Biomedical and Clinical Sciences, University Hospital “Luigi Sacco” Università di Milano, 20157 Milan, Italy; ^2^Scientific Institute, IRCCS Eugenio Medea, 23842 Bosisio Parini, Lecco, Italy; ^3^Pharmaceutical Service, Azienda Sanitaria Locale di Bergamo, 24100 Bergamo, Italy; ^4^Family Pediatricians, Territorial Health Service of Bergamo, 24100 Bergamo, Italy

## Abstract

We present the case of a 7-year-old boy, who received acetaminophen for the treatment of hyperpyrexia, due to an infection of the superior airways. 13 mg/kg (260 mg) of acetaminophen was administered orally before bedtime, and together with the expected antipyretic effect, the boy experienced sleep disruption and proprioceptive delirium. The symptoms disappeared within one hour. In the following six months, acetaminophen was administered again twice, and the reaction reappeared with similar features. Potential alternative explanations were excluded, and analysis with the Naranjo algorithm indicated a “probable” relationship between acetaminophen and this adverse reaction. We discuss the potential mechanisms involved, comprising imbalances in prostaglandin levels, alterations of dopamine, and cannabinoid and serotonin signalings.

## 1. Introduction

Acetaminophen (paracetamol) is the most widely used analgesic and antipyretic medicine, and it is found in many over-the-counter products. Although this drug has an excellent therapeutic index, in children, it is commonly associated with adverse drug reactions (ADRs), some of which are serious [1].

## 2. Case Presentation

We report on an ADR to acetaminophen occurred in a 7-year-old boy, affected by hyperpyrexia (38°C) due to an infection of the upper airways. He received acetaminophen 13 mg/kg (260 mg) orally before bedtime. Sleep was disturbed, and, after one hour, he awoke with a temperature of 37.5°C and manifested proprioceptive delirium, described as lengthening of the limbs. The symptoms disappeared within one hour. In the following six months, acetaminophen was administered again twice, at the same dose, and the reaction reappeared with a similar pattern and temporal profile. The intense proprioceptive alterations reported were in one case body swelling and in the other exploding and painful burning of the extremities. The pediatrician excluded hyperpyrexia as the cause of this reaction, since body temperature reached a maximum of 38°C, which was insufficient to cause neurological disturbances. Except for recurrent infections of the upper airways, ascribable to seasonal illness, the patient was in overall good health and never suffered from psychiatric disturbances. Considering these elements, analysis with the Naranjo ADR probability scale indicated the present adverse reaction to acetaminophen as “probable," an inference reinforced by positive repetitive dechallenges and re-challenges (detailed Naranjo score: +2 because the event appeared after administration of the suspect drug; +1 for positive dechallenge; +2 for positive rechallenge. All the other questions scored 0, total: 5).

## 3. Discussion

In the literature, there is only one case of psychosis related to the administration of acetaminophen plus codeine in an adult [2]. In the Italian Pharmacovigilance Registry, there are several cases of psychiatric adverse reactions to acetaminophen, all occurred in children and due to acetaminophen alone (maximum administered dose 500 mg). Four cases involved hallucinations, similar to the proprioceptive delirium here reported, and among these, two were accompanied by psychomotor agitation, while two different cases involved transient losses of consciousness. Although acetaminophen is not acting like a nonsteroidal anti-inflammatory drug, it can inhibit both the constitutive and inducible cyclooxygenase enzymes (COX1 and COX2), leading to a reduction in the production of prostaglandins (PG). This mechanism depends on an oxidative level-dependent inhibition of PGH synthase and occurs especially in the endothelium of the central nervous system (CNS). There is evidence that the cerebrovascular fluid levels of PGD2 and PGE2 regulate the onset and maintenance of sleep [3], implying that lowered levels of these prostaglandins lead to the alteration of sleep duration and to a loss of sleep architecture. An imbalance in PG synthesis has also been associated with delirium [4]. PGE2 reduces the levels of dopamine in the CNS, and its lack has been implicated in the pathogenesis of psychotic symptoms [5]. Interestingly, the frontal regions of the brain, strongly involved in psychotic phenomena, are especially permeable to acetaminophen [5], and this may contribute to explain why acetaminophen led to the ADR here reported. The acetaminophen metabolite N-arachidonyl-phenolamine has also been shown to activate the cannabinoid receptors implicated in the onset of delirium [6]. It also cannot be excluded that the effects of acetaminophen on the bulbospinal serotonin pathways may increase psychotic-like symptoms in peculiar conditions.

This is the first report of sleep disturbances and delirium due to acetaminophen, with a mechanistic hypothesis involving its effects on the prostaglandin, cannabinoid, and serotonin systems. Notably, the downstream effects of acetaminophen on the prostaglandin system may account for both sleep disruption and altered dopamine levels. Our results, together with the reactions previously reported in our country, demonstrate that unknown adverse drug reactions to acetaminophen, such as the psychiatric ones, are becoming increasingly reported. This suggests caution when using acetaminophen in children, especially in view of its widespread use.
